# Experimental Study and Numerical Analysis of the Seismic Performance of Glass-Fiber Reinforced Plastic Tube Ultra-High Performance Concrete Composite Columns

**DOI:** 10.3390/ma16216941

**Published:** 2023-10-29

**Authors:** Xiaopeng Tan, Mingqiao Zhu, Wanli Liu

**Affiliations:** 1 School of Civil Engineering, Hunan University of Science and Technology, Xiangtan 411100, China; xtyeeg@gmail.com (X.T.);; 2 Hunan Wisdom Construction Assembly Passive House Engineering Technology Research Center, Xiangtan 411100, China

**Keywords:** GFRP tube, UHPC, finite element analysis, seismic performance, shear capacity

## Abstract

To investigate the impact of the filament winding angle of glass-fiber reinforced plastic (GFRP) on the seismic behavior of GFRP tube ultra-high performance concrete (UHPC) composite columns, this study designs two types of GFRP tube UHPC composite columns. Quasi-static tests are conducted on the specimens subjected to horizontal reciprocating load and axial force, and the skeleton curve characteristics of the structure are analyzed. Furthermore, a finite element analysis model of the composite column is established to explore the effects of the diameter-thickness ratio, circumferential elastic modulus of confined tubes, and tensile strength of concrete on the seismic performance of the composite column. The analysis includes a review of the skeleton curve, energy dissipation capacity, and stiffness degradation of the structure under different designs. The results indicate that the use of GFRP tubes effectively enhances the seismic performance of UHPC columns. The failure mode, peak load, and peak displacement of the composite columns are improved. The finite element analysis results are in good agreement with the experimental results, validating the effectiveness of the analysis model. Extended analysis reveals that the bearing capacity of the specimen increases while the energy dissipation capacity decreases with a decrease in the diameter-thickness ratio and an increase in the circumferential elastic modulus. Although the tensile strength of concrete has some influence on the seismic performance of the specimen, its effect is relatively small. Through regression analysis, a formula for shear capacity suitable for GFRP tube UHPC composite columns is proposed. This formula provides a theoretical reference for the design and engineering practice of GFRP tube UHPC composite columns.

## 1. Introduction

In 1994, Larrard [1] pioneered the development of a concrete cube test block with an impressive compressive strength of 165 MPa, marking the inception of ultra-high performance concrete (UHPC). Since then, UHPC has gained significant traction and has found extensive applications in diverse fields such as construction engineering, bridge engineering, highway pavement, and maintenance [2,3]. The remarkable mechanical properties, durability, and corrosion resistance exhibited by UHPC have propelled the widespread utilization of fiber-reinforced polymer (FRP) in a wide range of civil structures, including buildings, bridges, highways, marine engineering projects, water conservancy initiatives, and military applications [4,5]. It is worth noting that the integration of UHPC and FRP has revolutionized the construction industry, offering enhanced structural performance, extended service life, and increased sustainability. The successful implementation of these advanced materials has contributed significantly to the advancement and resilience of various infrastructure systems.

The concrete-filled FRP tube (CFFT) column is an innovative structural system that combines the advantages of concrete and FRP tubes to enhance the strength and ductility of columns by effectively confining the transverse expansion of concrete. In comparison to concrete-filled steel tubes and reinforced concrete structures, FRP tube-confined concrete composite columns exhibit superior heat resistance, corrosion resistance, and insulation properties. The CFFT system has garnered significant attention from researchers, and numerous scholarly studies have been conducted in this area. Mirmiran et al. [6] conducted a comprehensive investigation into the axial compression behavior of FRP-tube concrete columns, providing a detailed analysis of the stress mechanism. The confinement effect of the FRP tube on the concrete delays the failure of the column, thereby enhancing the overall member’s durability, load-bearing capacity, and ductility [7]. Teng et al. conducted an extensive review of the current state of research on FRP fabric reinforcement for reinforced concrete structures. Their study encompasses various structural elements and examines the damage modes that occur after reinforcement. Additionally, the article presents a comprehensive overview of existing literature on load-bearing capacity models proposed for FRP reinforcement [8]. Marcin Abramski et al. carried out a study on the axial compressive performance of concrete columns restrained by GFRP tubes at three different angles, resulting in plots of longitudinal and peripheral deformations of polymer shells as a function of load level for both empty and concrete-filled tubes [9]. Lam and Teng conducted an analysis by summarizing the results of repeated compression tests on FRP-confined concrete conducted prior to 2009. Based on their findings, they proposed a design-oriented constitutive model for concrete confined by FRP that accurately predicts the response under repeated compression and unloading scenarios [10].

Currently, there are various combinations of concrete-filled FRP tube (CFFT) columns that have been studied. Cao et al. derived a stress–strain model for FRP-constrained rubber-concrete columns considering the cross-sectional shape through theoretical derivation [11], and Liu et al. carried out an experimental study on the axial compression performance of FRP-steel-concrete composite columns, and two types of failure modes were observed [12]. Fang et al. proposed a design using recycled aggregate concrete-filled glass fiber-reinforced polymer-steel composite tube columns. This design effectively addresses the structural deficiencies of recycled aggregate concrete (RAC) and includes an empirical model for predicting the transverse moment-displacement skeleton curve of concrete-filled GFRP-steel composite tube columns [13]. Togay Ozbakkalog et al. investigated the seismic behavior of high-strength concrete columns confined by fiber-reinforced polymer (FRP) tubes. Their research demonstrated that the inelastic deformation behavior of high-strength concrete (HSC) columns can be significantly improved by utilizing FRP tubes [14]. Feng et al. conducted an experimental study on the seismic behavior of fiber-reinforced polymer (FRP)-confined concrete core columns (FCCC). The results showed that these columns exhibit good energy dissipation and ductility, even under high axial loads [15]. Zhang et al. performed seismic tests on seven longitudinally reinforced elliptical carbon fiber-reinforced concrete (CFRC) columns. The study investigated the effects of elliptical aspect ratio, FRP thickness, and transverse cyclic load direction. Additionally, a numerical model was developed to simulate the seismic performance of these columns [16]. Fakharifar et al. proposed an FRP–PVC confined concrete column and conducted experimental research and theoretical analysis on its behavior. The study revealed that the PVC tube has a limited restraining effect on the concrete column, but it can withstand significant plastic deformation to accommodate concrete expansion. The wrapping of GFRP effectively restrains the local buckling of the PVC tube and the lateral expansion of the core concrete [17]. To improve the failure mode of concrete-filled steel tubes, Yu et al. utilized GFRP casing to confine the concrete-filled steel tubes and conducted quasi-static tests on composite column specimens with equal proportions. The results showed that the GFRP casing effectively delays or even prevents end buckling failure, thereby enhancing the flexural strength, ultimate horizontal bearing capacity, ductility, and energy dissipation capacity of the specimens [18]. Osama Youssf et al. proposed a rubber-clastic concrete composite column confined by CFRP and conducted experimental studies on its seismic behavior. The findings indicated that, compared to ordinary concrete columns confined by CFRP, the peak strength of the rubber-clastic concrete columns under CFRP confinement is higher [19].

In existing research, the influence of steel bars in the specimens on seismic performance is more significant compared to external GFRP. Additionally, most studies on the effects of parameters such as the thickness and strength of the confined tube on the seismic performance of composite columns are qualitative analyses. There is a lack of a unified standard for the practical application of engineering design methods, and limited research has been conducted on the seismic performance of GFRP tube UHPC composite columns. In order to study the essential factors affecting the seismic performance of this type of structure, the seismic performance of GFRP tube UHPC composite columns is investigated through experimental testing and finite element analysis. This study aims to verify the test results and expand the parameter analysis. The effects of the diameter-thickness ratio, circumferential elastic modulus of the confined tubes, and horizontal bearing capacity of concrete tensile strength specimens are thoroughly examined. Mathematical models are developed to provide guidance for designers in practical applications.

## 2. Experimental Study

### 2.1. Specimen Design

**Figure 1:**
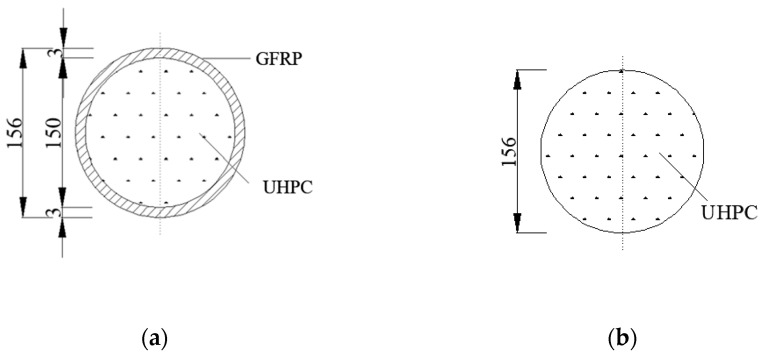
Section diagram of the specimen (**a**) GFRP tube UHPC composite column cross-section. (**b**) Cross-section of the UHPC comparison column.

In order to design longitudinal reinforcements for lateral resistance materials more reasonably, it is necessary to discuss the stress mechanism of UHPC materials under triaxial compression and seismic loads. Therefore, this study has investigated the seismic performance of GFRP tube UHPC composite columns, providing experimental and theoretical foundations for future research. Two composite columns with GFRP tubes and one UHPC column without GFRP tubes were designed and fabricated. The cross-section of the specimen is depicted in Figure 1, and the detailed parameters of the specimens are presented in Table 1.

There are two *θ* kinds of GFRP tubes used in the test, which are 45° and 80°, respectively. The inner diameter d of all composite columns is 150 mm, the wall thickness is *t* = 3 mm, and the length *l* is 1500 mm.

**Figure 2:**
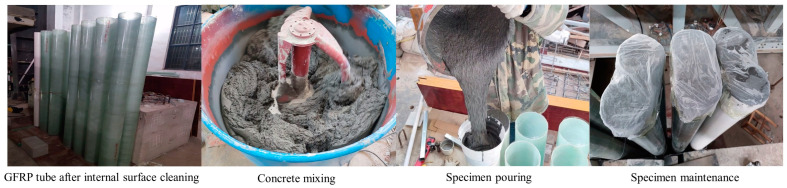
Fabrication process of UHPC-filled GFRP tube columns.

Each specimen was fabricated following the subsequent steps: Firstly, the inner surface of the GFRP tube required for the test was meticulously cleaned to eliminate any dust or oil residues, ensuring a tight interface between the GFRP and concrete. The concrete mixture was prepared using a JW300 forced concrete mixer(Zhengzhou Far East Machinery Manufacturing Co. Zhengzhou, China), where the measured ultra-high-performance concrete dry mixture, water, and admixtures were thoroughly mixed to form a cohesive gel. Subsequently, the mixture was poured into the GFRP tube through a funnel and conduit (a PVC tube was employed as a mold for the reference column specimen) to ensure the compactness of the concrete placement. Once the pouring was completed, the top of each specimen was covered with plastic film to maintain moisture, and regular watering was performed for proper curing. The manufacturing process of the specimens is illustrated in Figure 2.

### 2.2. Test Materials

The GFRP tube utilized in this experimental study is a filament wound tube manufactured by a reputable glass fiber-reinforced plastic company based in Guangdong. The filament winding process involves angles of 45° and 80°. The concrete employed in the test is C130UHPC powder, which is produced by Guangdong Guangzhou New Material Technology Co., Ltd (China). This concrete formulation possesses an expansion rate of 0.02%, enabling it to fulfill the requirements for micro-expansion and self-compaction. The UHPC mix ratio used in the experiment is as follows: C130 dry mixture to admixture to water ratio of 50:0.98:3.17, respectively.

#### 2.2.1. UHPC

##### Cube Compressive Strength

**Figure 3:**
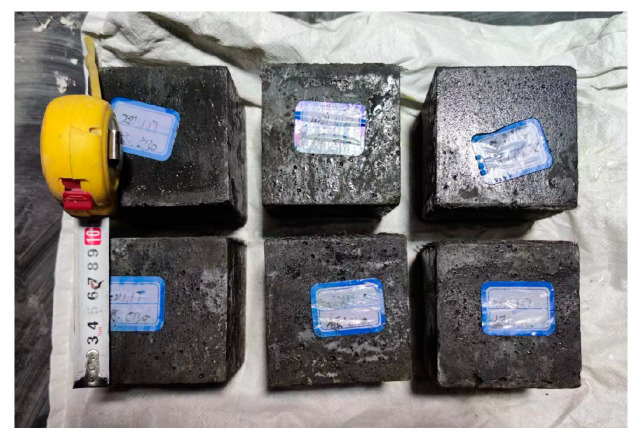
Cubic specimen.

In accordance with the prescribed curing conditions for the specimen, a total of six non-standard concrete cubes (100 mm × 100 mm × 100 mm) were cast, as illustrated in Figure 3. These cubes were subjected to compressive property testing, following the guidelines outlined in the GB/T 50081-2019 standard [20]. The resulting mechanical properties of the UHPC are presented in Table 2. It is important to note that the average value mentioned refers to the calculated mean obtained after excluding the maximum and minimum values.

Based on the formula proposed by YU and Ding [21], the compressive strength of standard cube blocks $f_{cu}$, UHPC cylinder compressive strength $f_{c}$, and UHPC modulus of elasticity $E_{c}$ is calculated according to the following formula:

(1) $$f_{cu}=1.17f_{cu,10}^{0.95}-0.7$$

(2) $$f_{c}=0.4f_{cu}^{7/6}$$

(3) $$E_{c}=9500f_{cu}^{1/3}$$

where $f_{cu,10}^{}$ is the strength of a cube specimen with a side length of 10 cm.

##### Cube Splitting Tensile Strength

**Figure 4:**
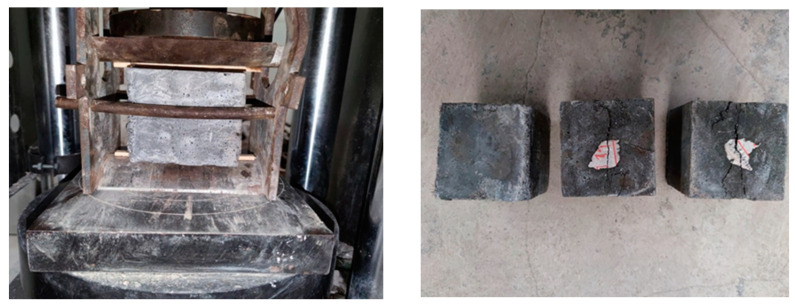
Splitting tensile test.

In accordance with the specified curing conditions for the specimen, three non-standard concrete cubes (100 mm × 100 mm × 100 mm) were cast, as depicted in Figure 4. The splitting tensile strength of the UHPC cube was measured in accordance with the guidelines outlined in the GB/T 50081-2019 standard [20]. It is important to note that a size conversion coefficient of 0.85 was applied to the 100 mm cube. The experimental results obtained from these tests are presented in Table 3.

(4) $$R_{ts}=\frac{2F}{\pi A}$$

where $R_{ts}$ is the cube splitting tensile strength (Mpa), *F* is the cube failure load (kN), and *A* is the cube splitting area (mm^2^).

#### 2.2.2. GFRP

**Figure 5:**
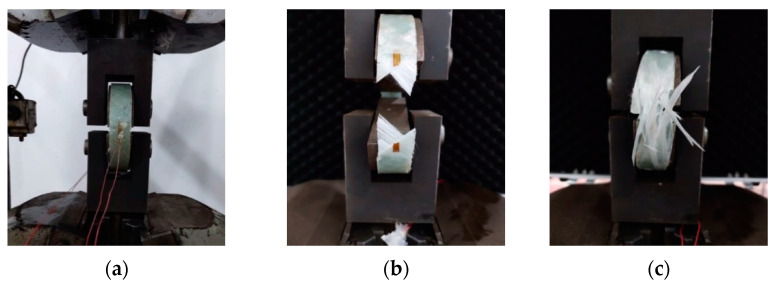
GFRP tube tensile test and test results. (**a**) Experimental equipment. (**b**) 45° specimen. (**c**) 80° specimen.

To ensure effective constraints, prefabricated filament-wound glass fiber-reinforced polymer (GFRP) tubes are utilized, with fibers oriented at 45° and 80° relative to the longitudinal axis. In order to evaluate the performance, ring tension tests are conducted following the guidelines specified in ASTM D2290-19 [22]. The tests involve three GFRP tubes with a wall thickness of 3 mm and a height of 30 mm. The test setup and the fractured GFRP ring are illustrated in Figure 5. The experimental results obtained from these tests are presented in Table 4.

### 2.3. Experimental Setup and Loading Scheme

**Figure 6:**
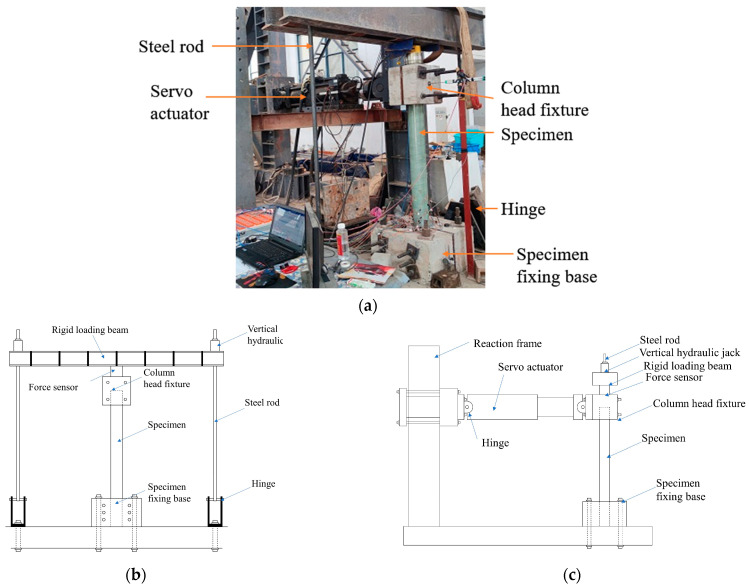
Experimental setup. (**a**) Experimental diagram. (**b**) Schematic diagram of the vertical loading device. (**c**) Schematic diagram of the horizontal loading device.

As depicted in Figure 6, we have designed and fabricated a precise loading device for our experimental setup. The lower end of the specimen is inserted into a dedicated hole beneath the fixed base. By utilizing screws positioned at both ends of the fixed base, the specimen is securely fastened to the base, which, in turn, is firmly affixed to the rigid laboratory floor using screws at the upper end. Figure 6b illustrates the vertical loading device, which comprises a fixed base specifically designed for the specimen, two hinged brackets, two high-strength steel tie rods, a rigid loading beam, a jack, and a pressure transducer. The axial force is applied using a jack with a measuring range of 100 t. Additionally, a pressure transducer with a measuring range of 2000 kN is employed to continuously monitor the axial force. To ensure accurate measurements and increase friction, the pressure transducer is placed on a column head fixture with quartz sand positioned between the transducer and the fixture, guaranteeing a level surface. Figure 6c showcases the transverse loading device, which consists of a computer-controlled electro-hydraulic servo actuator, a reaction frame, and a custom-designed fixture that securely attaches to the head of the column. The upper end of the specimen is connected to the MTS actuator via the upper fixture. The loaded end of the actuator is ball-hinged to the upper fixture of the specimen, while the other end is bolted to the reaction frame, ensuring the actuator maintains a horizontal position throughout the experiment.

**Figure 7:**
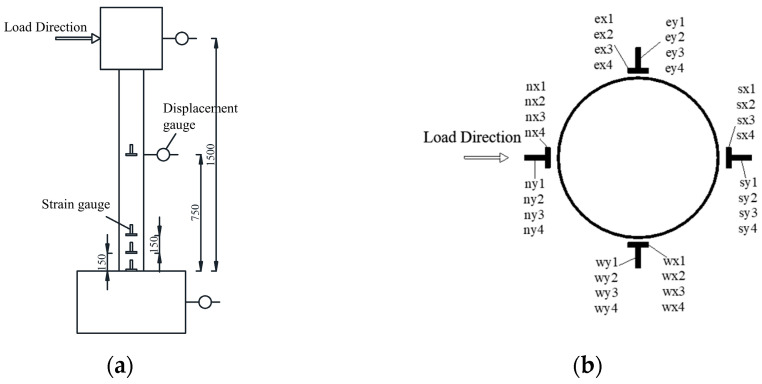
Layout of displacement gauges and strain gauges. (**a**) Displacement gauge. (**b**) Strain gauges.

To capture the deformation of the specimen during the entire loading process, three displacement acquisition points have been established. These points are placed to monitor specific aspects of the specimen’s behavior. Displacement Gauge 1: Located at the fixed base of the specimen, this gauge is intended to monitor any potential sliding or movement of the base during the loading process. It helps ensure the stability of the specimen’s foundation. Displacement Gauge 2: Positioned at a height of 750 mm along the column, this gauge is employed to observe the bending deformation of the specimen as it undergoes loading. It provides insights into the specimen’s flexural behavior. Displacement Gauge 3: Placed at the horizontal displacement loading point, this gauge is utilized to monitor the horizontal displacement of the loading point. It helps track any lateral movement or displacement that occurs during the loading process; this is also compared to the actuator displacement output data. For data acquisition, resistive displacement gauges are used at each of these three points. These gauges are designed to measure and record displacement values accurately. The arrangement of the displacement gauges can be referred to in Figure 7a.

In the low-cycle reciprocating loading test of the column, the column foot area is typically the location where failure occurs. Therefore, to capture strain data accurately, strain gauges are placed at specific heights along the column. The arrangement includes four locations: 0 mm, 150 mm, 300 mm, and 750 mm. At each height, both longitudinal and transverse strain gauges are applied. Additionally, four groups of strain gauges are evenly distributed along the circumference of the column. In total, there are 32 strain gauges used in one specimen. The specific arrangement can be referred to in Figure 7b.

**Figure 8:**
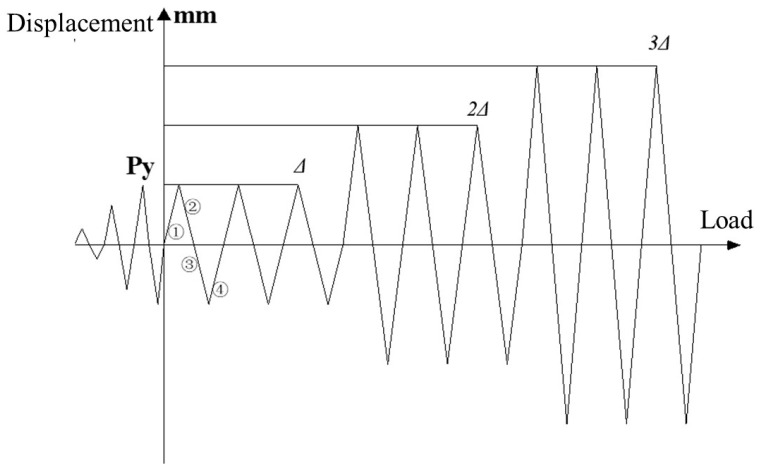
Schemes for the cyclic lateral loading. Where ① is the 1th lateral formal loading, and so on.

In the test procedure, the axial force is initially preloaded in a graded manner. The preloading axial force is set at 60% of the actual axial force, which is 120 kN. Throughout the test, the rationality of loading is assessed based on observation data, and the axial force is gradually increased until it reaches the predetermined level. After applying the axial force, horizontal loading is conducted using a displacement control method. The loading scheme follows the guidelines outlined in JGJ/T101-2015 [23]. The horizontal loading is initially carried out with smaller displacements, with each stage of displacement being 1 mm. Each stage of displacement is cyclically loaded once. During the test, the specimen’s behavior is monitored. When a significant decrease in the stiffness of the specimen is detected, the value of the yield displacement (∆) at this point is recorded. At this point, the specimen enters the yielding stage, and the yield displacement (∆) is recorded. The displacements applied are in the order of ∆, 2∆, and 3∆, with a total of three cycles. The experiment concludes when the peak horizontal load of the specimen decreases to 85% or less of the maximum peak horizontal load. The specific horizontal loading system can be referred to in Figure 8.

### 2.4. Experimental Results and Discussion

#### 2.4.1. Failure Modes

**Figure 9:**
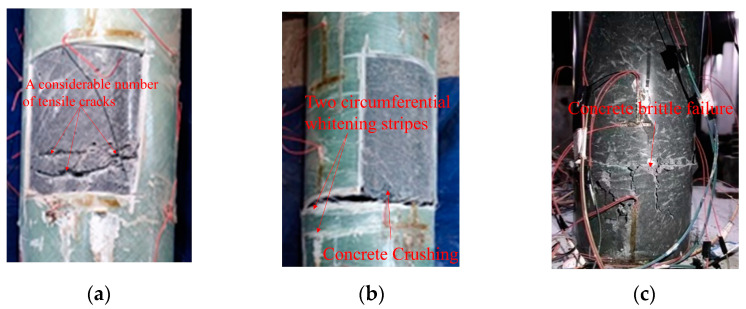
Failure mode of specimens. (**a**) FC45-2. (**b**) FC80-2. (**c**) C-2.

Based on the observations in Figure 9, it is evident that the GFRP tube of the FC45-2 specimen exhibited significant whitening over a 12 cm section. It is important to note that GFRP materials exhibit a distinctive failure mode known as whitening. Under compressive loads, the walls of GFRP pipes gradually deform, leading to the initiation of fiber fractures within the material. These fractured fibers reflect and scatter light, resulting in a whitening or opaqueness of the GFRP pipe surface. Therefore, the occurrence of whitening in GFRP pipes can be indicative of achieving or approaching failure. No other noticeable damage was observed. Upon cutting the GFRP tube in the failure area of the specimen, a considerable number of tensile cracks were discovered within the internal concrete, resulting in concrete crushing. In the case of the FC80-2 specimen, two circumferential whitening stripes were observed along the filament winding angle at a height of 2 cm from the bottom column. When the GFRP tube in the failure area of this specimen was cut, only the edge section of the concrete exhibited complete crushing failure. In contrast, the contrast column specimen, which lacked the wrapping of a GFRP tube, displayed a horizontal circumferential crack at the bottom and at a height of approximately 10 cm during the later stages of loading. Subsequently, the concrete continued to peel off, exhibiting brittle failure. These observations suggest that the presence of a GFRP tube provides enhanced structural integrity, as evidenced by the absence of significant damage in the GFRP-wrapped specimens compared to the contrast column specimen.

#### 2.4.2. Skeleton Curves

**Figure 10:**
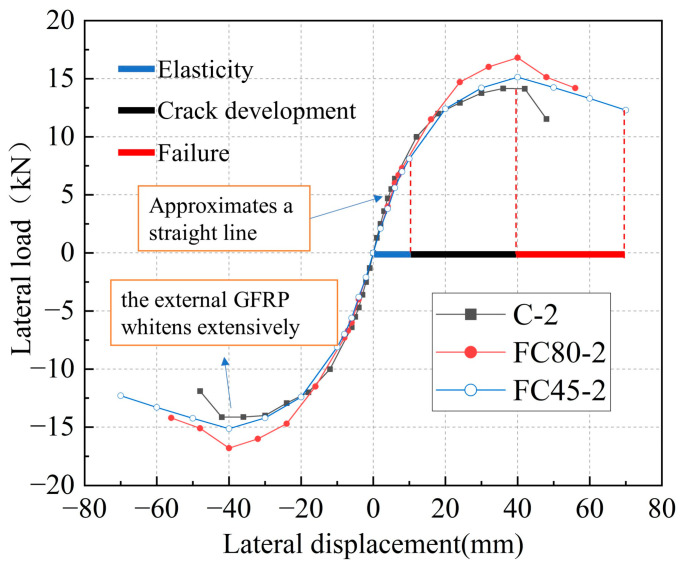
Skeleton curves of specimens.

In Figure 10, the horizontal cyclic loading process of the three types of specimens can be divided into three stages: the elastic stage, the crack development stage, and the failure stage. In the initial stage of loading (within a range of +/− 20 mm), the skeleton curve of the specimens approximates a straight line, but different specimens have different horizontal loads at the same displacement, indicating differences in their initial stiffness. At this stage, the structure undergoes elastic deformation. As the displacement increases, the specimens emit slight noises, and the concrete inside the structure expands. White stripes appear on the surface of the GFRP tube in the direction of fiber winding. The GFRP tube provides confinement, and the internal concrete begins to develop initial cracks, marking the crack development stage. When the loading displacement reaches a certain level, the external GFRP turns white over a large area, indicating a decrease in confinement capacity and an intensification of core concrete damage. Eventually, the specimen loses its load-bearing capacity and enters the failure stage.

By comparing the ultimate displacements of the two types of GFRP-reinforced UHPC composite columns with the ultimate displacement of the UHPC column without GFRP confinement, it is evident that the former has a larger displacement. However, due to the limited number of test samples and budget constraints that prevented repetitive testing, a comprehensive analysis of the seismic performance of GFRP-reinforced UHPC composite columns could not be conducted. Therefore, this study aims to conduct finite element analysis on the test models to gain an in-depth understanding of the seismic performance of composite structures and propose rational design methods.

## 3. Numerical Simulations

### 3.1. Materials Constitutive Models

#### 3.1.1. GFRP Tube

The damage to the GFRP tube is significantly influenced by the dislocation of fibers and the shear behavior of the resin, which poses challenges in studying its behavior [8]. Previous experimental studies have indicated that FRP materials demonstrate linear elastic behavior and limited plastic deformation under tension. Therefore, for the purpose of this study’s finite element analysis, it is assumed that the GFRP tube is an orthotropic material that exhibits linear elastic deformation in each direction. In finite element modeling and analysis using ANSYS17.2 software, according to the range given in the help manual, specific parameters such as elastic modulus, shear modulus, Poisson’s ratio, and ultimate tensile strength of GFRP materials are defined. The values of specific parameters are shown in Table 5.

#### 3.1.2. UHPC

Referring to Li et al. [24], the complete stress–strain curve equations for ultra-high performance concrete under uniaxial compression versus uniaxial tension are as follows:

(5) $$y=\left\{\begin{array}{c} 1.18x+0.1x^{5}-0.28x^{6}\;0\leq x\leq 1\\ \frac{x}{8.5{\left(x-1\right)}^{2}+x}\;x>1 \end{array}\right.\;$$

where $x=\varepsilon_{c}/\varepsilon_{co}$, $\varepsilon_{c}$ is the concrete strain, $\varepsilon_{co}$ is the concrete peak strain, $y=\sigma_{c}/f_{c}$, $\sigma_{c}$ is the concrete stress, $f_{c}$ is the prism axial compressive strength, according to the experimental results of this material property.

(6) $$y=\left\{\begin{array}{c} 1.12x+0.76x^{2}-0.88x^{3}\;0\leq x\leq 1\\ \frac{x}{8.2{\left(x-1\right)}^{1.7}+x}\;x>1 \end{array}\right.$$

where $x=\varepsilon_{t}/\varepsilon_{to}$, $y=\sigma_{t}/\sigma_{t0}$. $\varepsilon_{to}$ is the peak strain corresponding to the tensile stress–strain curve of concrete, $\sigma_{t0}$ is the stress value corresponding to the peak strain of the tensile stress–strain curve of concrete, according to the experimental results of this material property.

**Figure 11:**
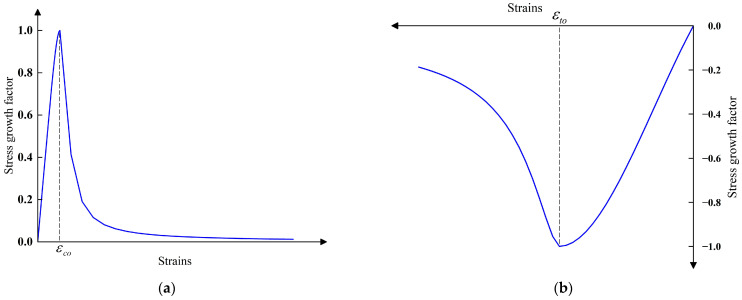
Tensile and compressive stress–strain diagrams for UHPC. (**a**) UHPC compressive stress–strain modeling. (**b**) UHPC tensile stress–strain modeling.

Figure 11 shows the tensile and compressive stress–strain diagrams for UHPC.

### 3.2. Finite Element Model Validation

It is mentioned that the ANSYS program provides various material models for finite element modeling of GFRP tubes and concrete. In this study, the linear elastic behavior of the GFRP tube is defined using the BISO model in the software. Additionally, the high-strength concrete model confined by the GFRP tube is defined based on an improved Drucker–Prager criterion proposed by the research group [25].

The model in this study is established based on the C-2, FC45-2, and FC80-2 specimens tested. Two basic assumptions need to be met:

1. There is no relative slip between the GFRP pipe and the concrete.
2. The stressing process satisfies the conditions of internal and external force balance and longitudinal deformation coordination.

**Figure 12:**
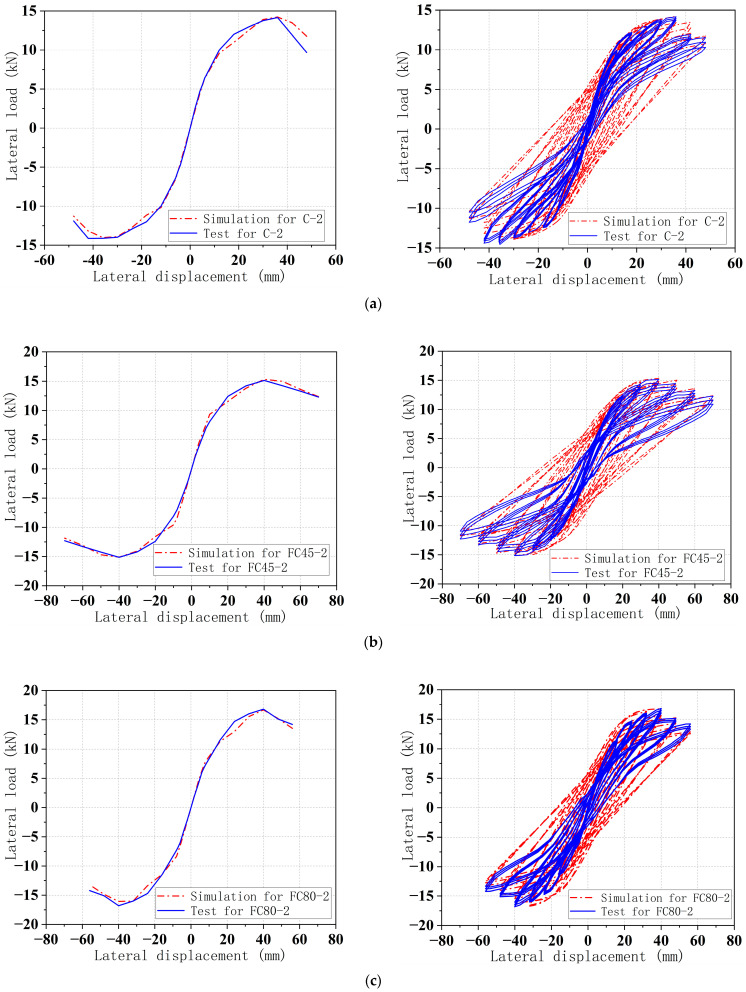
Comparisons of skeleton curves between tests and simulations. (**a**) specimen C-2. (**b**) specimen FC45-2. (**c**) specimen FC80-2.

The obtained skeleton curve and full test curves from the test are compared with the numerical simulation results, as shown in Figure 12. Furthermore, the comparison between the test and the simulated bearing capacity is presented in Table 6.

The analysis results demonstrate good agreement with the test results, as indicated in Table 6. The maximum horizontal bearing capacity shows a small error, and the test results are consistent with the decrease in positive and negative directional loads. The ultimate bearing capacity and the slope of the descending section of the skeleton curve also align well with the experimental results.

There is a difference in the early stiffness of the specimen. This is mainly manifested by the fact that at the beginning of loading, the load values of the simulation results are higher than the test results for the same displacements. Further analysis reveals that the ideal loading conditions used in the finite element simulation do not accurately capture the gap and slip between the specimen and the loading device in the test. This discrepancy results in a higher stiffness in the early stages of the simulation curve. Based on the above analysis, it can be concluded that the material constitutive relation utilized in the finite element analysis effectively reflects the mechanical characteristics of the GFRP tube UHPC composite column under quasi-static testing. Additionally, it can also capture the hysteresis curve of the specimen under different parameters to analyze the energy dissipation capacity of the specimen’s stiffness degradation, thus verifying the effectiveness of the finite element simulation.

## 4. Expanded Parameter Analysis

To ensure the accuracy of both the test and simulation results and to minimize the influence of other factors, a total of 16 specimens were designed using data from the FC45-2 and FC80-2 models. In consideration of practical engineering applications, the seismic performance of these specimens was analyzed in relation to the diameter-thickness ratio, circumferential elastic modulus of the confined tubes, and tensile strength of the concrete. Notably, the discussion on the circumferential elastic modulus of the confined tubes takes into account the commonly used types of FRP materials in structural applications. It is worth mentioning that the majority of circumferential elastic moduli of confined tubes, with various FRP materials and processing methods, fall within the range of 20 to 200 GPa [26]. The design parameters of the specimens are presented in Table 7.

### 4.1. Skeleton Curve Analysis

**Figure 13:**
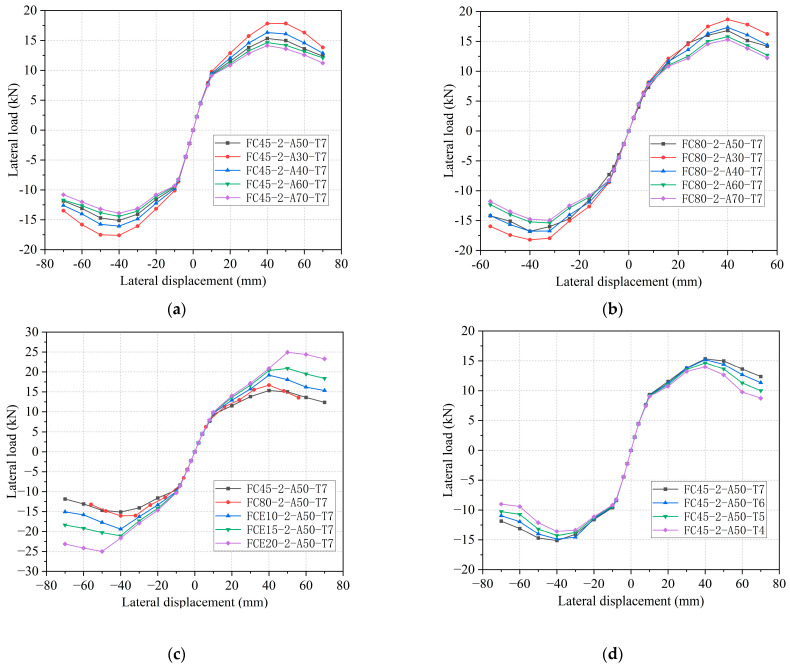
Comparisons of skeleton curves. (**a**) Different diameters of 45° GFRP tubes. (**b**) Different diameters of 80° GFRP tubes. (**c**) Restraint tubes of different strengths. (**d**) Different tensile strengths of concrete.

The skeleton curve provides a macroscopic representation of the seismic performance of each characteristic point (data acquisition points) of the specimen, including displacement, load, ductility, strength degradation, and stiffness degradation. By correlating the specimen data with parameters such as the diameter-thickness ratio, circumferential elastic modulus of the restrained tube, and concrete tensile strength, the skeleton curve depicted in Figure 13 was generated. From Figure 13, it can be observed that for specimens with a GFRP fiber 45° winding angle, when the diameter-thickness ratio is set at 30, 40, 60, and 70, the peak bearing capacity of the specimen is measured at 17.81 kN, 16.32 kN, 14.65 kN, and 14.14 kN, respectively. Compared to the FC45-2-A50-T7-S20 specimen with a diameter-thickness ratio of 50, the variation range of the diameter-thickness ratio is −40%, −20%, 20%, and 40%, resulting in changes of 16.2%, 6.5%, −4.4%, and −7.7%, respectively. Similarly, for specimens with a GFRP fiber 80° winding angle, when the diameter-thickness ratio is set at 30, 40, 60, and 70, the peak bearing capacity of the specimen is measured at 18.67 kN, 17.34 kN, 15.78 kN, and 15.31 kN, respectively. Compared to the FC80-2-A50-T7-S20 specimen with a diameter-thickness ratio of 50, the variation range of the diameter-thickness ratio is −40%, −20%, 20%, and 40%, resulting in changes of 11.9%, 3.9%, −5.4%, and −8.3%, respectively. When the diameter-thickness ratio decreases, the peak load of the specimen increases while the peak displacement remains unaffected.

When the circumferential elastic modulus of the constrained tube is set to 54 GPa, 100 GPa, 150 GPa, and 200 GPa, the corresponding peak bearing capacities of the specimen are measured at 16.73 kN, 19.18 kN, 20.88 kN, and 24.92 kN, respectively. In comparison to the FC45-2-A50-T7 specimen with a circumferential elastic modulus of 29 GPa, the peak bearing capacity exhibits a variation range of 9.4%, 25.1%, 36.2%, and 62.6%, respectively. It is evident that an increase in the circumferential elastic modulus of the restrained tube leads to an increase in the peak load displacement and peak load of the specimen.

The peak displacement of the specimen is primarily influenced by specimen damage. For specimens with different diameter-thickness ratios and varying concrete tensile strengths, the peak displacement consistently remains at 40 mm. This indicates that an increase in GFRP thickness has a minimal impact on delaying the damage to the core concrete. However, when the circumferential elastic modulus of the restrained tube reaches a certain level, the peak displacement of the FCE20-2-A50-T7-S20 specimen increases from 40 mm to 50 mm. A restrained tube with a higher elastic modulus can effectively restrain the crushed concrete under larger displacements, thereby delaying specimen damage. Additionally, a smaller diameter-thickness ratio results in a steeper decreasing trend after reaching the peak load, while an increase in the circumferential elastic modulus slows down the downward trend of the specimen after reaching the peak load. Therefore, it can be concluded that decreasing the diameter-thickness ratio and increasing the circumferential elastic modulus can delay the damage to the core concrete during the intermediate loading stage, thereby improving the seismic performance of the specimen. However, in the later loading stage, the restraining effect caused by increased thickness rapidly diminishes due to the damage to the GFRP tube, whereas the increase in circumferential elastic modulus continues to provide persistent benefits. In practical engineering applications, the method of increasing the circumferential elastic modulus of restrained tubes should be prioritized to enhance the seismic performance of composite columns.

When the concrete tensile strength of the 45° angle specimen is set at 6 MPa, 5 MPa, and 4 MPa, the corresponding peak bearing capacities of the specimen are measured at 15.2 kN, 14.65 kN, and 13.98 kN, respectively. Compared to the FC45-2-A50-T7-S20 specimen with a concrete tensile strength of 7 MPa, the peak bearing capacity exhibits a change range of −0.8%, −4.4%, and −8.8%, respectively. As the tensile strength of the concrete decreases, the peak bearing capacity of the specimen also decreases. Following the peak load, the decreasing trend becomes steeper, although it does not significantly affect the peak displacement of the specimen.

### 4.2. Degradation of Stiffness

**Figure 14:**
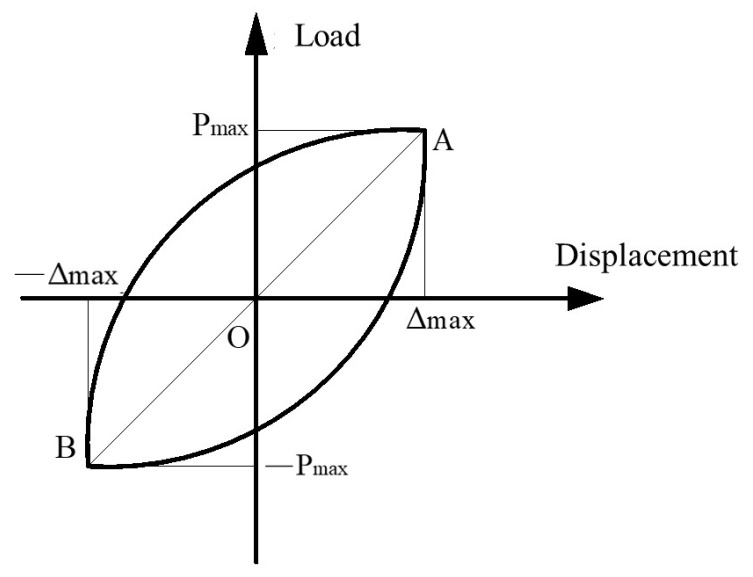
Schematic diagram of equivalent stiffness calculation. Where A,B are the peak points of positive and negative loads.

In this paper, the equivalent stiffness [27] is used to describe the stiffness change. The equivalent stiffness is calculated as shown in Figure 14. The equivalent stiffness is that the positive and negative peak points of the hysteretic curve of the first cycle of each stage are connected by AB, and the slope of the AB connection line is the equivalent stiffness.

(7) $$K_{i1}=\frac{\left|P_{i1A}\right|+\left|P_{i1B}\right|}{\left|\Delta_{i1A}\right|+\left|\Delta_{i1B}\right|}$$

where *K_i_*_1_ is the equivalent stiffness of stage *i* loading cycle; *P_i_*_1*A*_ is the positive peak load of stage *i* loading the first cycle; *P_i1B_* is the negative peak load of stage *i* loading the first cycle; Δ*_i_*_1*A*_ is the positive peak displacement of stage *i* loading the first cycle; Δ*_i_*_1*B*_ is the negative peak displacement of stage *i* loading the first cycle; *i* is the loading series.

**Figure 15:**
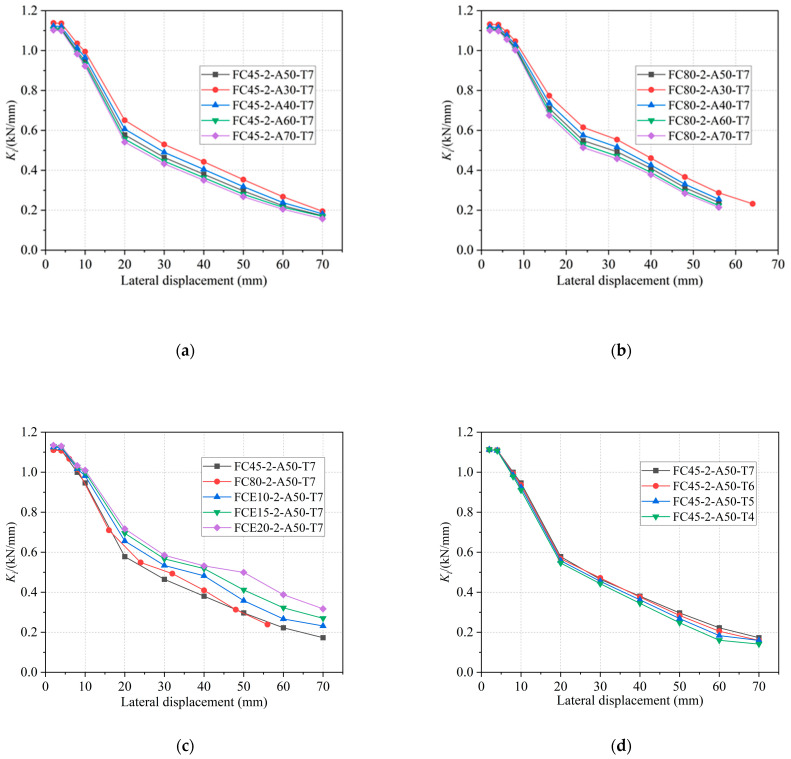
Comparisons of secant stiffness degradations. (**a**) Different diameters of 45° GFRP tubes. (**b**) Different diameters of 80° GFRP tubes. (**c**) Restraint tubes of different strengths. (**d**) Different tensile strengths of concrete.

The above calculation results are shown in Figure 15 to discuss the effects of the diameter-thickness ratio, the circumferential elastic modulus of the restrained tube, and the tensile strength of concrete on the stiffness degradation of the specimen.

Figure 15a,b demonstrates that the specimens with two different winding angles exhibit an increase in equivalent stiffness as the diameter-thickness ratio decreases. Initially, the difference in stiffness between the specimens increases, but it subsequently decreases with the progression of loading displacement. However, overall, when varying the diameter-thickness ratio, the Secant stiffness curve of the specimens does not exhibit significant differentiation. Referring to Figure 15c,d, it is evident that a higher circumferential elastic modulus of the restrained tube results in greater specimen stiffness. Furthermore, the stiffness degradation curve becomes more pronounced in the later stages of loading. The irregular fluctuation of the stiffness of the specimens in the later stage may be due to the varying degrees of damage to the internal concrete, and the effect of stronger constraints on the stiffness is nonlinear, but the overall trend is not affected. Specimens with a larger circumferential elastic modulus of the restrained tube experience a slower attenuation of stiffness. Conversely, the tensile strength of concrete does not exert a noticeable effect on the stiffness of the specimens.

### 4.3. Energy Dissipation Capacity

**Figure 16:**
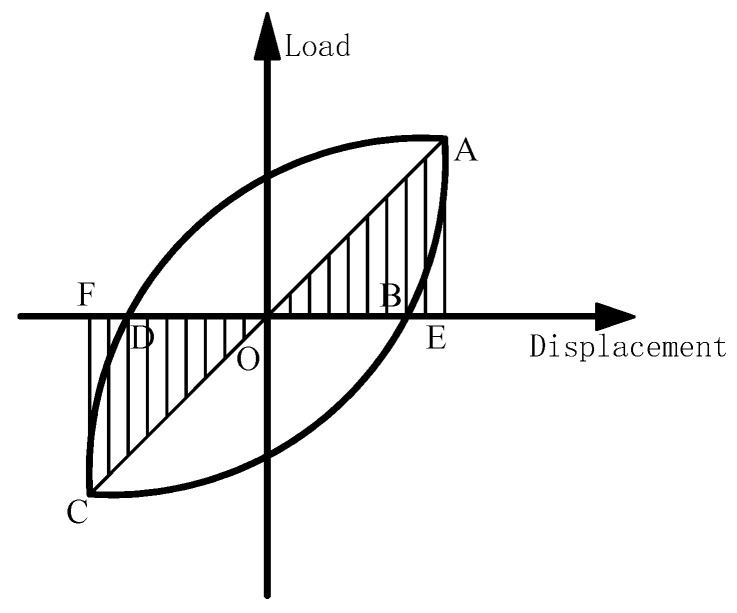
Schematic diagram of energy consumption coefficient calculation. Where A, C is the peak point of positive and negative loads, E, F are the intersection of A, C and the vertical line of the coordinate axis, B, D are the intersection of the hysteresis loop and the coordinate axis.

The energy dissipation capacity of the composite column specimens studied in this paper can be analyzed with reference to the wrapping area of the hysteretic curve. With reference to the building seismic test code [28], the energy dissipation coefficient *E* can be used to evaluate the energy dissipation capacity of the specimen. The calculation diagram is shown in Figure 16, and the calculation method is as follows:

(8) $$E=\frac{S_{\left(ABC+CDA\right)}}{S_{\left(OEA+OFC\right)}}$$

**Figure 17:**
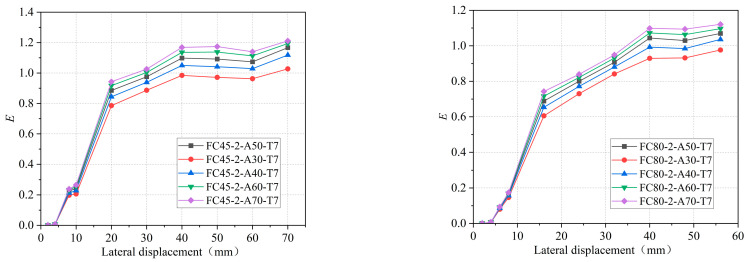

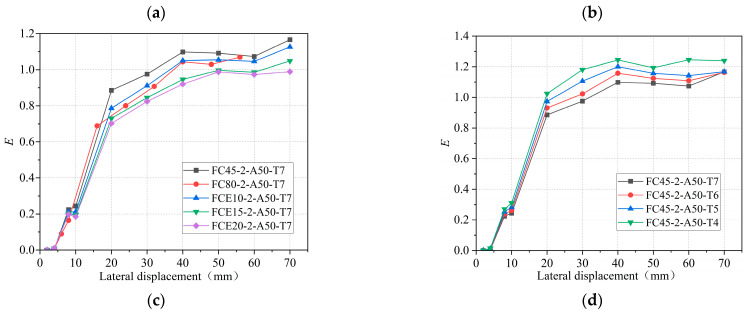
Comparisons of energy consumption capacity. (**a**) Different diameters of 45° GFRP tubes. (**b**) Different diameters of 80° GFRP tubes. (**c**) Restraint tubes of different strengths. (**d**) Different tensile strengths of concrete.

The energy consumption coefficient of each specimen is obtained, as shown in Figure 17.

Figure 17 reveals several observations regarding the energy dissipation capacity of the specimen. Firstly, as the diameter-thickness ratio decreases, the energy dissipation capacity also decreases. Additionally, the energy dissipation capacity decreases with an increase in both the circumferential elastic modulus of the restrained tube and the tensile strength of the concrete. This phenomenon can be attributed to the increased constraint on the internal concrete when the thickness of the external GFRP tube and the circumferential elastic modulus of the restrained tube increase. Consequently, the damage to the concrete decreases proportionally. Similarly, when the tensile strength of the concrete increases, the extent of tensile damage to the internal concrete diminishes, leading to an increase in specimen strength and a decrease in energy dissipation capacity. Furthermore, the energy dissipation coefficient of the specimen increases with each loading cycle, indicating a gradual increase in energy dissipation capacity. However, as the loading displacement increases, the rate of increase in energy dissipation capacity initially decreases and then begins to rise again. During the initial stage of loading, the restraint effect of the GFRP tube on the micro-cracking and expansion of concrete gradually intensifies. As a result, the proportion of elastic deformation to total deformation increases, leading to a gradual decrease in the rate of increase in energy dissipation capacity. In the final loading stage, the external GFRP tube loses its restraint ability and undergoes destruction. This intensifies the damage to the core concrete, causing it to contribute the largest proportion to the total deformation and exhibit the least elastic deformation. Consequently, the increase in energy dissipation capacity becomes more significant during this stage.

## 5. Seismic Load Bearing Capacity Formula

For the calculation of the seismic bearing capacity of GRFP tube confined UHPC composite columns in this experiment, with reference to the derivation process of the formula for calculating the shear capacity of concrete-filled steel tube in the codes GB50936-2014 [29] and JIN [30], the contribution of GFRP tube, axial force, and core concrete to the shear capacity of GFRP tube UHPC composite columns is calculated based on the superposition principle, and the total bearing capacity $V_{u}$ is:

(9) $$V_{u}=V_{f}+V_{c}+V_{n}$$

In the formula, $V_{f}$, $V_{c}$ and $V_{n}$ are, respectively, the contribution of GFRP tube, core concrete, and axial force to the bearing capacity. When the shear span ratio *m* > 3, the contribution of the GFRP tube to the shear capacity of columns is generally bending failure. In this case, reference is made to the contribution of steel tubes in concrete-filled steel tubes to shear capacity [31,32]. Compared with the specimens in this paper, the contribution of GFRP tubes to the shear capacity of composite columns is $V_{f}$:

(10) $$V_{f}=\frac{1+\alpha_{s}{}^{2}}{6m}f_{y}A_{f}$$

where $\alpha_{s}$ is the ratio of concrete section width to GFRP tube section width, *m* is the shear-span ratio of composite columns,$A_{f}$ is the cross-sectional area of GFRP tube, and $f_{y}$ is the ultimate strength of GFRP.

For the contribution of axial force *N* to the shear capacity of composite columns, $V_{n}$ is obtained by using the shear transfer mechanism of the arch mechanism and assuming that the height of the compression zone is *h*/2 [33].

(11) $$V_{n}=N\tan\varphi =\frac{1}{4m}N$$

For the contribution of core concrete to the shear capacity of composite columns, refer to the code [29], $V_{c}$ is expressed as:

(12) $$V_{c}=\frac{\alpha }{m+1}f_{t}A_{c}$$

To sum up, the formula for the shear capacity of GFRP tube UHPC composite columns is as follows:

(13) $$V_{u}=\beta \frac{1+\alpha_{s}{}^{2}}{6m}f_{y}A_{f}+\frac{1}{4m}N+\frac{\alpha }{m+1}f_{t}A_{c}$$

where *m* is the shear-span ratio,α is the coefficient of partial shear capacity of concrete,$f_{t}$ is the design value of concrete tensile strength, and $\alpha_{s}$ is the ratio of concrete section width to GFRP tube section width.

Based on the above formula, using the numerical simulation data, regression analysis and correction are carried out. For GRFP tube-confined UHPC composite columns, the following formula is suggested to calculate the shear capacity:

(14) $$V_{u}=\beta \frac{1+\alpha_{s}{}^{2}}{6m}f_{y}A_{f}+\frac{1}{4m}N+\frac{0.75}{m+1}f_{t}A_{c}$$

According to the experimental results and finite element analysis, the relationship between the coefficient β and the constraint strength introduced in the regression analysis is as follows:

(15) $$\beta =0.027\ln(\frac{Et}{60})+0.1241$$

In order to verify the correctness of the formula, specimen analyses for the FC45-2 and FC80-2S specimens and the 16 parameters in Table 7 were calculated using Equation (14) based on the experimental data of similar specimens by scholars [34]. The calculated results are shown in Table 8.

*V_s_* is the maximum shear capacity measured in the experiment, and *V_g_* is the calculated value of the shear capacity formula obtained by regression analysis in this paper.$V_{s}/V_{g}$: Average value $\bar{X}$ = 1.020, sample variance *s*^2^ = 0.0032, sample standard deviation *s* = 0.057, coefficient of variation δ = 0.056. To sum up, the formula for calculating the shear capacity of GRFP tube-confined UHPC composite columns proposed in this paper has high accuracy and universality and can be used to calculate the shear capacity of this type of composite column.

## 6. Conclusions

This paper focuses on investigating the seismic performance of GFRP tube-confined UHPC composite columns through a quasi-static test with an axial compression ratio of 0.2. A refined numerical analysis model is established to further understand the mechanical characteristics of the composite structure, and a rational calculation method for seismic bearing capacity is proposed. Based on the research conducted, the following conclusions can be drawn:

- The GFRP tube has a significant positive impact on enhancing the seismic performance of UHPC. It not only increases the peak load and peak displacement of the column but also effectively improves the failure pattern of the composite structure, preventing concrete fragmentation and peeling phenomena.
- A finite element analysis model for GFRP tube-confined UHPC composite columns is proposed. Through parameter analysis, it is observed that reducing the diameter-thickness ratio and increasing the circumferential elastic modulus enhance the bearing capacity of the specimen. However, these changes result in a decrease in energy dissipation capacity. Overall, an increase in the circumferential elastic modulus of the confined tube exhibits a more significant effect and demonstrates good persistence. Specimens with higher tensile strength of concrete exhibit larger bearing capacities but worse energy dissipation capacity. In addition, expanding the current database of GFRP tube UHPC composite column tests would significantly contribute to further substantiating the proposed conclusions.
- By utilizing expanded parameter finite element analysis data and test data, a seismic bearing capacity formula for GFRP tube-confined UHPC composite columns is derived through regression and validated against test results and experimental data from other researchers. The proposed formula aligns well with the results, demonstrating high accuracy and universality. It serves as a valuable reference for designers.

## Figures and Tables

**Table 1:** Specimen details.

Specimen	*d*/mm	*t*/mm	*θ/*(*°*)	*l*/mm	*n*
C-2	156	0	0	1500	0.2
FC45-2	150	3	45	1500	0.2
FC80-2	150	3	80	1500	0.2

Where *θ* is the angle of the GFRP fibers to the *Y*-axis during winding and forming, and *n* is the axial pressure ratio.

**Table 2:** Specimen test values and calculations.

Serial Number	Measured Value (MPa)	Average Value (Mpa)	Standard Deviation (Mpa)	$f_{cu}$ (Mpa)	$f_{c}$ (Mpa)	$E_{c}$ (Mpa)
1	110.5	109.6	5.04	100.7	86.9	44.29
2	108.0					
3	102.7					
4	105.6					
5	113.2					
6	118.1					

**Table 3:** Splitting tensile test results.

Serial Number	1	2	3	Average Value	Standard Deviation	*f_ts_*
Splitting tensile dissociation/MPa	11.3	11.5	13.2	12.0	0.85	10.2

**Table 4:** GFRP tube tensile test results.

Serial Number		Tensile Strength (MPa)	Modulus of Elasticity (Gpa)
45° specimen	1	79.34	28.71
	2	81.91	29.64
	3	79.44	28.75
	Average value	80.23	29.03
	Standard deviation	1.19	0.43
80° specimen	1	426.82	54.02
	2	428.18	54.2
	3	428.85	54.28
	Average value	427.95	54.17
	Standard deviation	0.84	0.11

**Table 5:** The parameters of GFRP materials.

*θ*/°	*E*_1_/GPa	*E*_2_/Gpa	*V*_1_	*V*_2_	*G_XY_*/Mpa	*G_YZ_*/Mpa	*G*_XZ_/Mpa
45	29.03	3.41	0.2	0.21	2300	1300	1300
80	54.17	2.53	0.2	0.21	6000	1600	1600

Note: *θ* is the filament winding angle of the GFRP tube, *E*_1_ and *E*_2_ are the elastic moduli of the GFRP tube in the circumferential and axial direction; *V*_1_ and *V*_2_ are the Poisson’s ratios of the GFRP tube in the circumferential and axial direction; and *G_XY_*, *G_YZ,_* and *G_XZ_* are the corresponding shear moduli of subscript.

**Table 6:** Comparison of experimental and simulated bearing capacities.

	FC45-2	FC80-2	C-2
Test load capacity *P*_1_ (kN)	15.13	16.48	14.13
Simulated Load Capacity *P*_2_ (kN)	15.33	16.73	14.22
*P*_1_/*P*_2_	0.987	0.985	0.994

**Table 7:** Component design parameters.

Specimen Number	*θ* (*E*/MPa)	*t* (mm)	Diameter to Thickness Ratio	$f_{t}$ (MPa)
FC45-2-A30-T7	45° (29,000)	5	30	7
FC45-2-A40-T7	45° (29,000)	3.75	40	7
FC45-2-A50-T7	45° (29,000)	3	50	7
FC45-2-A60-T7	45° (29,000)	2.5	60	7
FC45-2-A70-T7	45° (29,000)	2.14	70	7
FC80-2-A30-T7	80° (54,000)	5	30	7
FC80-2-A40-T7	80° (54,000)	3.75	40	7
FC80-2-A50-T7	80° (54,000)	3	50	7
FC80-2-A60-T7	80° (54,000)	2.5	60	7
FC80-2-A70-T7	80° (54,000)	2.14	70	7
FCE10-2-A50-T7	100,000	3	50	7
FCE15-2-A50-T7	150,000	3	50	7
FCE20-2-A50-T7	200,000	3	50	7
FC45-2-A50-T6	45° (29,000)	3	50	6
FC45-2-A50-T5	45° (29,000)	3	50	5
FC45-2-A50-T4	45° (29,000)	3	50	4

Note: For the sake of uniform naming, specimen FC45-2 is renamed as FC45-2-A50-T7, and specimen FC80-2 is renamed as FC80-2-A50-T7. Taking FCE10-2-A50-T7 as an example, FCE10 means that the peripheral elasticity of the restrained pipe is 1,000,000 MPa, 2 means that the axial compression ratio is 0.2, and A50 means that the diameter-thickness ratio is 50 m T7, and the concrete tensile strength is 7 MPa.

**Table 8:** Comparison analysis of experimental values and calculated values.

Specimen Number	$V_{s}$ (kN)	$V_{g}$ (kN)
FC45-2-A30-T7-S20	17.705	16.798
FC45-2-A40-T7-S20	16.190	15.726
FC45-2-A50-T7-S20	15.215	15.125
FC45-2-A60-T7-S20	14.540	14.746
FC45-2-A70-T7-S20	14.020	14.486
FC80-2-A30-T7-S20	18.445	19.067
FC80-2-A40-T7-S20	17.055	17.337
FC80-2-A50-T7-S20	16.375	16.356
FC80-2-A60-T7-S20	15.585	15.733
FC80-2-A70-T7-S20	15.135	15.303
FCE10-2-A50-T7-S20	19.180	18.956
FCE15-2-A50-T7-S20	20.880	21.664
FCE20-2-A50-T7-S20	24.920	24.515
FC45-2-A50-T6-S20	15.200	15.045
FC45-2-A50-T5-S20	14.650	14.402
FC45-2-A50-T4-S20	13.980	13.760
Literatures [34] RCFF-0	28.269	30.530

## Data Availability

Not applicable.

## References

[1] De Larrard F., Sedran T.J.C. (1994). Optimization of ultra-high-performance concrete by the use of a packing model. Cem. Concr. Res..

[2] Tanaka Y., Maekawa K., Kameyama Y., Ohtake A., Musha H., Watanabe N. (2011). The innovation and application of UHPFRC bridges in Japan. Designing and Building with UHPFRC.

[3] Bierwagen D., Abu-Hawash A. Ultra high performance concrete highway bridge. Proceedings of the 2005 Mid-Continent Transportation Research Symposium.

[4] Yu T., Hu Y., Teng J.G. (2014). FRP-confined circular concrete-filled steel tubular columns under cyclic axial compression. J. Constr. Steel Res..

[5] Chen J. (2009). Special Issue FRP Composites in Construction. Constr. Build. Mater..

[6] Mirmiran A., Shahawy M.J. (1997). Behavior of concrete columns confined by fiber composites. J. Struct. Eng..

[7] Mukherjee A., Ramana V., Kant T., Dutta P., Desai Y. (1998). Behavior of Concrete Columns Confined by Fiber Composites. Discussion and Closure. J. Struct. Eng..

[8] Teng J.G., Chen J.F., Smith S.T., Lam L. (2003). Behaviour and strength of FRP-strengthened RC structures: A state of the art review. Buildings.

[9] Abramski M., Korzeniowski P., Klempka K.J.M. (2020). Experimental studies of concrete-filled composite tubes under axial short-and long-term loads. Materials.

[10] Lam L., Teng J.G. (2009). Stress–strain model for FRP-confined concrete under cyclic axial compression. Eng. Struct..

[11] Cao Y., Zhao G., Zhang Y., Hou C., Mao L.J. (2022). Unified Stress-Strain Model of FRP-Confined Square and Circle Rubber Concrete Columns. Materials.

[12] Liu J., Ma D., Dong F., Liu Z. (2023). Experimental Study on the Impact of Using FRP Sheets on the Axial Compressive Performance of Short-Circular Composite Columns. Materials.

[13] Fang S., Liu F., Xiong Z., Fang J., Li L.J. (2019). Seismic performance of recycled aggregate concrete-filled glass fibre-reinforced polymer-steel composite tube columns. Materials.

[14] Ozbakkaloglu T., Saatcioglu M. (2006). Seismic behavior of high-strength concrete columns confined by fiber-reinforced polymer tubes. J. Compos. Constr..

[15] Feng P., Cheng S., Yu T. (2018). Seismic performance of hybrid columns of concrete-filled square steel tube with FRP-confined concrete core. J. Compos. Constr..

[16] Zhang B., Zhou C., Wang C., Li Y., Gao Y., Zheng H.J. (2023). Seismic behavior and modeling of elliptical concrete-filled FRP tubes with longitudinal steel rebars under lateral cyclic load and vertical constant load. Thin-Walled Struct..

[17] Fakharifar M., Chen G.J.C. (2017). FRP-confined concrete filled PVC tubes: A new design concept for ductile column construction in seismic regions. Materials.

[18] Yu T., Hu Y., Teng J.G. (2016). Cyclic lateral response of FRP-confined circular concrete-filled steel tubular columns. J. Compos. Constr..

[19] Youssf O., ElGawady M.A., Mills J. (2016). Static cyclic behaviour of FRP-confined crumb rubber concrete columns. Eng. Struct..

[20] (2019). Test Method Standard for Physical and Mechanical Properties of Concrete.

[21] Yu Z.W., Ding J. (2003). Uniform method of calculating mechanical properties of concrete under compression. J. Build. Struct..

[22] (2019). Standard Test Method for Apparent Hoop Tensile Strength of Plastic or Reinforced Plastic Pipe by Split Disk Method.

[23] (2015). Seismic Testing Regulations for Buildings.

[24] Li L., Zheng W.C., Lu J.H. (2010). An experimental study on the mechanical properties of activated powder concrete. J. Harbin Inst. Technol..

[25] Wang L.J., Zhu M.Q., Dong J.R. (2023). Uniaxial compression analysis model of GFRP pipe-constrained high-strength concrete short columns based on improved Drucker-Prager criterion. J. Appl. Mech..

[26] Amran Y.M., Alyousef R., Rashid R.S., Alabduljabbar H., Hung C.C. (2018). Properties and applications of FRP in strengthening RC structures: A review. Structures.

[27] (2010). Code for Design of Concrete Structures.

[28] (1996). Code of Practice for Seismic Testing Methods for Buildings.

[29] (2014). Technical Specification for Steel Pipe Concrete Structures.

[30] Jin L., Fan L., Li D., Du X.J. (2020). Size effect of square concrete-filled steel tubular columns subjected to lateral shear and axial compression: Modelling and formulation. Thin-Walled Struct..

[31] Tomii M., Sakino K.J. (1979). Experimental studies on concrete filled square steel tubular beam-columns subjected to monotonic shearing force and constant axial force. Trans. Archit. Inst. Jpn..

[32] Ye Y., Han L.-H., Tao Z., Guo S.-L. (2016). Experimental behaviour of concrete-filled steel tubular members under lateral shear loads. J. Constr. Steel Res..

[33] Council A.T. (2009). Quantification of Building Seismic Performance Factors.

[34] Xiao J.Z., Huang Y.J. (2012). Seismic performance and damage evaluation of GFRP pipe-confined recycled concrete columns. J. Civ. Eng..

